# Relevance of Biofilms in the Pathogenesis of Shiga-Toxin-Producing *Escherichia coli* Infection

**DOI:** 10.1155/2013/607258

**Published:** 2013-11-12

**Authors:** Natalia Angel Villegas, José Baronetti, Inés Albesa, Rosana Polifroni, Alberto Parma, Analía Etcheverría, Maria Becerra, Nora Padola, Maria Paraje

**Affiliations:** ^1^IMBIV, CONICET y Departamento de Farmacia, Facultad de Ciencias Químicas, Universidad Nacional de Córdoba, Haya de la Torre y Medina Allende, 5000 Córdoba, Argentina; ^2^Laboratorio de Inmunoquímica y Biotecnología, Departamento de SAMP, Facultad de Ciencias Veterinarias, Universidad Nacional del Centro de la Provincia de Buenos Aires, 7000 Tandil, Argentina

## Abstract

The present study was designed to determine the relationships among biofilm formation, cellular stress and release of Shiga toxin (Stx) by three different clinical Shiga toxin-producing *Escherichia coli* (STEC) strains. The biofilm formation was determined using crystal violet stain in tryptic soy broth or thioglycollate medium with the addition of sugars (glucose or mannose) or hydrogen peroxide. The reactive oxygen species (ROSs) were detected by the reduction of nitro blue tetrazolium and reactive nitrogen intermediates (RNI) determined by the Griess assay. In addition, the activities of two antioxidant enzymes, superoxide dismutase (SOD) and catalase (CAT), were studied. For the cytotoxicity studies, Vero cells were cultured with Stx released of STEC biofilms. The addition of sugars in both culture mediums resulted in an increase in biofilm biomass, with a decrease in ROS and RNI production, low levels of SOD and CAT activity, and minimal cytotoxic effects. However, under stressful conditions, an important increase in the antioxidant enzyme activity and high level of Stx production were observed. The disturbance in the prooxidant-antioxidant balance and its effect on the production and release of Stx evaluated under different conditions of biofilm formation may contribute to a better understanding of the relevance of biofilms in the pathogenesis of STEC infection.

## 1. Introduction

 Hemorrhagic colitis, which occasionally progresses to hemolytic uremic syndrome (HUS) in children and other susceptible groups of individuals, is a hallmark of human infection with Shiga toxin-producing *Escherichia coli* (STEC). *Escherichia coli* O157 : H7 is the serotype most commonly associated with clinical disease and food-associated outbreaks. However, other STEC serotypes such as O111 and O26 have also been associated with outbreaks and sporadic disease [1, 2]. Two types of Shiga toxins (Stx), Stx1 and Stx2, are known and they constitute the main virulence factors in STEC strains [3–5]. Vero cells have a high sensitivity to Stx, and the cytotoxicity assay using this cell line is often used as the gold standard to evaluate diagnostic immunoassays. These cells have on their plasma membrane receptors a high concentration of Gb3 and Gb4, thus allowing detection of Stx1, Stx2, and its variants. STEC strains released toxins into the culture medium and the specific cytotoxicity on Vero cells is used to determine the ability of bacterial isolates from clinical specimens and Stx producing food. Stx2 toxin is produced and released into the medium continuously during the exponential growth phase, while Stx1 toxin accumulates in the periplasmic space of the bacteria and it released at the end of the exponential phase [6, 7].

Biofilms have a microbial multicellular lifestyle, and they are defined as organized communities of bacteria collaborating among themselves and being attached to an inert or living surface contained in an extracellular polymeric substance (EPS) [8]. Biofilm STEC formation has been reported on stainless steel, glass, and polystyrene and it may be regarded as a survival strategy of bacteria such as *E. coli* O157 : H7 [9–11]. However, biofilms can cause serious problems in both medicine and industry, through increasing the resistance of cells to environmental stresses and protecting them from cleaning and sanitation procedures [10, 12].

Diverse stresses, including low nutrients and oxygen availability, high osmolarity, ethanol, and subinhibitory antibiotic concentrations, can alter the cellular functions associated with the oxidative metabolism, thereby stimulating the production of reactive oxygen species (ROS), including the highly reactive hydroxyl radicals (HO^∙^), hydrogen peroxide (H_2_O_2_), and the superoxide anion (O_2_^−^) [13]. Oxidative stress is caused by an imbalance between the productions of oxidants, such as the free radicals, and the levels of antioxidant defenses. A disturbance in the prooxidant-antioxidant balance in favor of the overproduction of ROS can result in damage to the cellular components. Another form of stress is termed nitrosative stress, where reactive nitrogen intermediates (RNI), such as nitrate (NO_3_^−^) and nitrite (NO_2_^−^), are used as terminal electron acceptors under anaerobic conditions, whereas nitric oxide (NO) is a short-lived free radical produced enzymatically by nitric oxide synthesis in various types of cells, and it has a great diffusion across membranes [14]. In the antioxidant defense system, some major enzymes involved in the detoxification of ROS are superoxide dismutase (SOD) and catalase (CAT), among others [15]. However, oxidative imbalance is due to an overproduction of ROS or a reduction in the oxidative defenses is insufficient to remove the free radicals, and therefore the antioxidant system plays a very important role in the control of this process.

Bacteria may also encounter extracellular fluxes of ROS from phagocytic cells during infection or when nonspecific oxidizing biocides are employed as disinfectants. Although adaptive responses against oxidative stress caused by these ROS have been extensively studied with planktonic cells, comparatively little is known about the biofilm responses. Biofilms have been shown to become increasingly resistant to repeated doses of antibiotics or nonspecific oxidizing biocides [16], but the basis for this apparent acquired resistance is currently unknown. We previously described a model system for examining the oxidative stress generated in a biofilm [13] but further studies are still necessary to determine the consequences of the imbalance between the production of oxidants and the levels of antioxidant defenses in the biofilms. The objective of the present study was to determine the relationships among STEC biofilm formation, cellular stress, and release of Stx under different culture conditions. To our knowledge, it is the first study that has attempted to correlate this biofilm formation with the disturbance in the prooxidant-antioxidant balance and its effect on the production and release of Stx. The study shows that the alteration of biofilm environment can be suitable for release of Stx and it could contribute to the understanding of the pathogenesis of infection by this pathogen.

## 2. Material and Methods

### 2.1. Bacterial Strains and Culture Conditions

The biofilm formation of *E. coli* O157 : H7 (strain N° 1-Stx1 and Stx2-) and *E. coli* O111 : H-(strain N° 2-Stx1-) clinical isolates (these strains produce Shiga-toxin and they were associated with HUS) and the reference strain *E. coli* EDL 933 (strain N° 3-Stx1 and Stx2-) were studied. Clinical isolates were kindly provided by the Microbiology Laboratory of the Pediatric Hospital of Córdoba, Provincia de Córdoba, Argentina [14, 17]. Stock cultures were preserved at −80°C using glycerol 15% (v/v) as the cryoprotectant, and the *E. coli* strains were grown in tryptic soy broth (TSB) at 37°C for 18 h. 

### 2.2. Quantification of Biofilm Assay Using a Microtiter Plate Assay

 The assay for the biofilm formation used in this study was adapted from the method of O'Toole and Kolter [18], which is based on the ability of bacteria to form biofilm on solid surfaces and it uses crystal violet (CV) to stain biofilms. In brief, 200 *μ*L of dilution 1/10 of overnight culture in TSB was added in each well of flat-bottomed microtiter plates (96-well, Greiner Bio-One, Germany), at 37°C for 24 h without shaking. After incubation, the supernatant was separated and the flat-bottomed microtiter plate was washed twice with phosphate buffer solution (PBS) pH 7.2. 

Diverse culture conditions were assayed using TSB alone, with added glucose (0.5%) or mannose (0.5%) [13, 19]. The influence of the reduction conditions was assayed in thioglycollate broth, and the microaerobic conditions were also studied [13]. TSB or thioglycollate was used as negative controls to obtain a background value. H_2_O_2_ is considered a major endogenous source of oxidative stress [20]. Influence of oxidative stress induced by exogenous application of H_2_O_2_ (Merck) was assayed in concentrations ranging from 2.5 to 30 mM and was added to each well with TSB.

Biofilm formation was investigated under static conditions at 37°C for 24 h. After incubation, the supernatant was separated for determining cellular stressand cytotoxicity effect and the plates were washed three times with 200 *μ*L of PBS. Then plates were air dried for 24 h prior to staining the adherent biofilms in each well and stained with 200 *μ*L of 1% (w/v) CV in water for 30 min. Finally, the plates were rinsed with very gently running distilled water until no stain was visible. The quantitative analysis of the biofilm production was performed by extracting the CV with 200 *μ*L per well of the bleaching solution: ethanol/glacial acetone (70 : 30), and the optical density (OD) was determined at 595 nm using a microplate reader (Model 680 BioRad, Hercules, CA). All strains were tested in three independent experiments on different days. 

The average OD from the control wells (ODc) was subtracted from the OD_595_ nm of all test wells. A microtiter plate biofilm assay was performed in triplicate for all strains, and the averages and standard deviations were calculated for all experiments. Strains were classified as follows: OD ≤ ODc = no biofilm producer; ODc < OD ≤ (2 × ODc) = weak biofilm producer; (2 × ODc) < OD ≤ (4 × ODc) = moderate biofilm producer; and (4 × ODc) < OD = strong biofilm producer [21]. The biofilm biomass unit (BBU) was arbitrarily defined with 0.1 OD_595_ equal to 1 BBU [13].

### 2.3. Assays for Oxidative Metabolites and Antioxidative Activity

The production of ROS was detected in the supernatant (0.1 mL) by the reduction of nitro blue tetrazolium (NBT-Sigma) to form an insoluble dark blue diformazan precipitate (0.1 mL of NBT 1 mg mL^−1^). The by-products of the assay are proportional to the ROS generated in biofilms and were measured at 540 nm [13, 22].

NO production was determined by the measurement of nitrite in cell free-supernatants using the Griess reaction. Briefly, Griess reagent was prepared by mixing equal volumes of sulfanilamide (1.5% in 1 N HCl) and N-(1-naphthyl)ethylenediamine dihydrochloride (0.13% in sterile distilled water). A volume of 200 *μ*L of Griess reagent was then mixed with 100 *μ*L of sample aliquots, and this mixture incubated for 15 min in the dark. The OD at 540 nm was measured with an automated microplate reader (Bio-Rad, Hercules, CA, USA), and the concentration of nitrite calculated from a NaNO_2_ standard curve [14, 22]. 

Total SOD activity was assayed photochemically based on the inhibition of NBT reduction. The ability of SOD to inhibit the reduction of NBT by the O_2_^−^ generated through the illumination of riboflavin in the presence of oxygen and the electron donor methionine was evaluated in the samples [15]. The results were expressed as SOD activity (%)/BBU.

To quantify CAT activity, the mature biofilms were treated with 50 *μ*L of PBS, 40 *μ*L of 0.2 M H_2_O_2_, and 200 *μ*L of 0.2 M potassium dichromate (K_2_Cr_2_O_7_) solution in glacial acetic acid. The curve of the reaction was made with different concentrations of pure CAT plus the reagents mentioned above. The OD was determined at 570 nm and the results were expressed as CAT (U)/BBU [16]. 

### 2.4. Vero Cell Cytotoxicity Assay

The cytotoxicity of culture supernatants was evaluated by Vero cells assay. Vero cells were grown at 37°C in Eagle's minimal essential medium (MEM) supplemented with 10% (vol/vol) fetal calf serum, 100 mg/liter penicillin, 200 mg/liter streptomycin, and 2.2 g/liter NaHCO_3_ in an atmosphere of 5% CO_2_. The supernatant of each strain and culture conditions was centrifuged at 17,228 ×g, 10 min at 4°C, filtered with 0.22 *μ*m membrane and 50 *μ*L of each one was inoculated in 96-well-plates containing 4 × 10^4^ freshly trypsinized Vero cells and incubated 48 h at 37°C in a 5% CO_2_ atmosphere. The cell monolayers were fixed with 10% (v/v) formaldehyde and then stained with 0.2% (w/v) CV in PBS [6, 7]. The cytotoxic effects were evaluated after 24 h by light microscopy. Then, for each sample, images from three randomly selected positions were obtained and analyzed using an Olympus Fluoview FV 1000. For image analysis, three investigators (N.A.V., I.A., and M.G.P.) evaluated the images independently in a blinded retrospective manner. Results are expressed as damage percentage (%) with respect to controls ± SD LPS. *E. coli* EDL 933 (strain N° 3) was used as positive control, and a strain Stx positive without cytotoxic effect was used as negative control (*E. coli* serotype O15 : H21) [23].

### 2.5. Statistical Analysis

All experiments were performed in triplicate, and numerical data are presented as means with error bars representing standard deviations. The data were statistically analyzed by using ANOVA followed by the Student-Newman-Keuls test for multiple comparisons. The differences between means were assessed with a *P versus TSB < 0.01 and ^#^ *P* versus thioglycollate medium < 0.01 being considered statistically significant. 

## 3. Results

### 3.1. Influence of Different Culture Conditions on Oxidant Metabolites and Antioxidant Defenses in Biofilms

**Figure 1:**
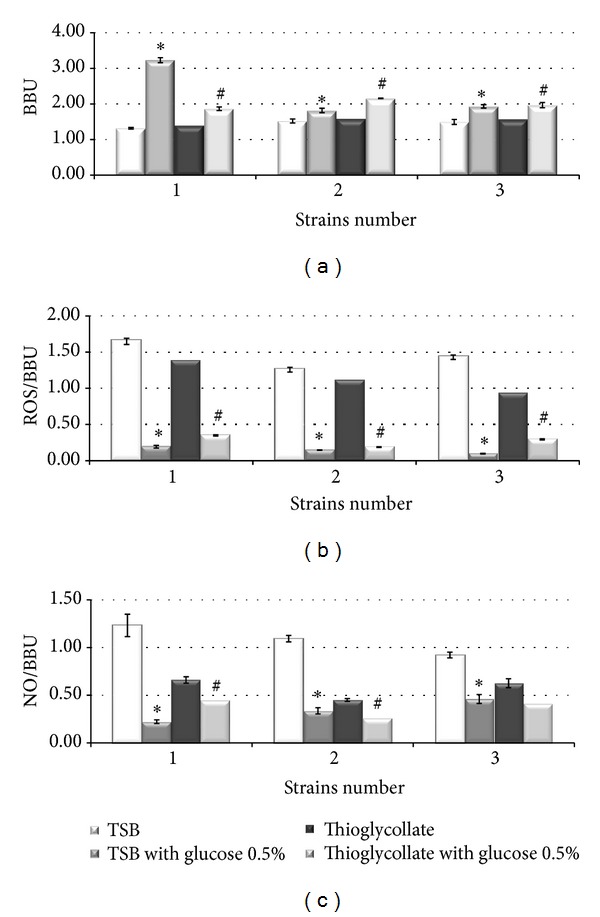
Quantification of biofilm formation of STEC strains by crystal violet (CV) staining expressed in biofilm biomass units (BBU): (a) in TSB; with addition of 0.5% glucose; in thioglycollate medium alone and with the addition of 0.5% glucose. (b) ROS ratio (ROS/BBU) determined by NBT assay. (c) NO and BBU ratio (NO/BBU) determined by Griess method. Error bars represent the standard deviations of the means of three independent experiments. ^#^ *P* versus TSB < 0.01 and ^#^ *P* versus thioglycollate medium < 0.01 being considered statistically significant.

A quantitative analysis of biofilm formation indicated that the three STEC strains showed “weak biofilm producer” (according to the scale described in Materials and Methods) biofilm formation in TSB (Figure 1(a)). When we studied the effect of adding 0.5% glucose to TSB on the production of biofilms, a significant increase in biofilm production “moderate biofilm producer” was seen in *E. coli* O157 : H7 (strain N° 1) (BBU = 1.31 ± 0.02 to 3.23 ± 0.07) and a smaller but still significant increase in both *E. coli* O111 (strain N° 2) (BBU = 1.52 ± 0.06 to 1.82 ± 0.06) and *E. coli* EDL 933 (strain N° 3) (BBU = 1.50 ± 0.07 to 1.93 ± 0.05) (*P  versus TSB < 0.01). Biofilm formation was increased similarly with the addition of mannose to TSB (data not shown). 

To evaluate other culture conditions in which the oxygen tension was smaller (reduction and atmospheric conditions), biofilms were grown in thioglycollate medium. The resulting values of BBU were for strain N° 1 (BBU = 1.41 ± 0.05), N° 2 (BBU = 1.59 ± 0.04), and N° 3 (BBU = 1.58 ± 0.03). No difference was observed between TSB and thioglycollate medium (*P versus TSB < 0.01). When assays were performed with thioglycollate medium in aerobiosis with the addition of glucose, an increase in biofilm formation was seen in strain N° 1 (BBU = 1.87 ± 0.05), N° 2 (BBU = 2.16 ± 0.07), and N° 3 (BBU = 1.97 ± 0.07) too (^#^ *P* versus thioglycollate medium < 0.01).

Data in Figure 1(b) indicate that STEC produced detectable amounts of ROS in the biofilms evaluated by NBT and these assays were useful in determining the relationship between ROS and the biofilm formation (BBU). When glucose was added in TSB medium, biofilm formation increased and the production of ROS was reduced, with an important 14-fold decrease observed in strain N° 3 and an 8-fold in the others. We also observed that when the assays were performed with thioglycollate medium, the biofilm formation resulted in less production of ROS compared to TSB and the glucose influence was not so markedly (3 to 6-fold) (^#^ *P* versus thioglycollate medium < 0.01).

The production of detectable amounts of RNI (NO) in the biofilm is shown in Figure 1(c). We found similar patterns of stress metabolites (ROS and NO) in the biofilms with the addition of glucose. When this medium was replaced by thioglycollate medium, a decrease of NO was also observed. 

**Figure 2:**
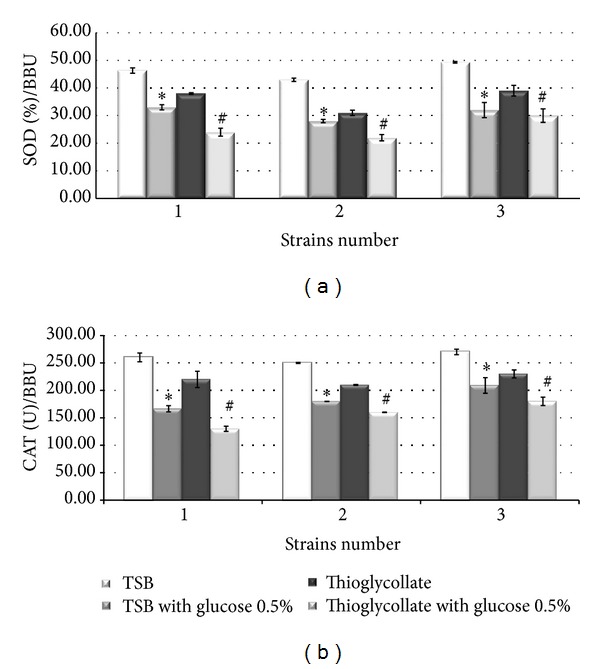
Antioxidant defenses in biofilms of STEC: (a) SOD activity (%)/BBU and (b) CAT (U)/BBU in TSB; with addition of 0.5% glucose; in thioglycollate medium and in thioglycollate medium with addition of 0.5% glucose. Each column shows the mean ± SEM of three independent experiments. *P < 0.01 respect to TSB and ^#^ *P* versus thioglycollate medium < 0.01.

The SOD and CAT activities were studied to attempt to correlate biofilm formation with changes in ROS and RNI production under different culture conditions (Figure 2). The SOD and CAT activity were decreased significantly in TSB with the addition of glucose and in thioglycollate medium and correlated with low levels of ROS. 

The total production of biofilm, oxidant metabolites, and antioxidant enzymes in TSB or thioglycollate medium was found to be approximately the same for both aerobic and microaerobic conditions (data not shown).

**Figure 3:**
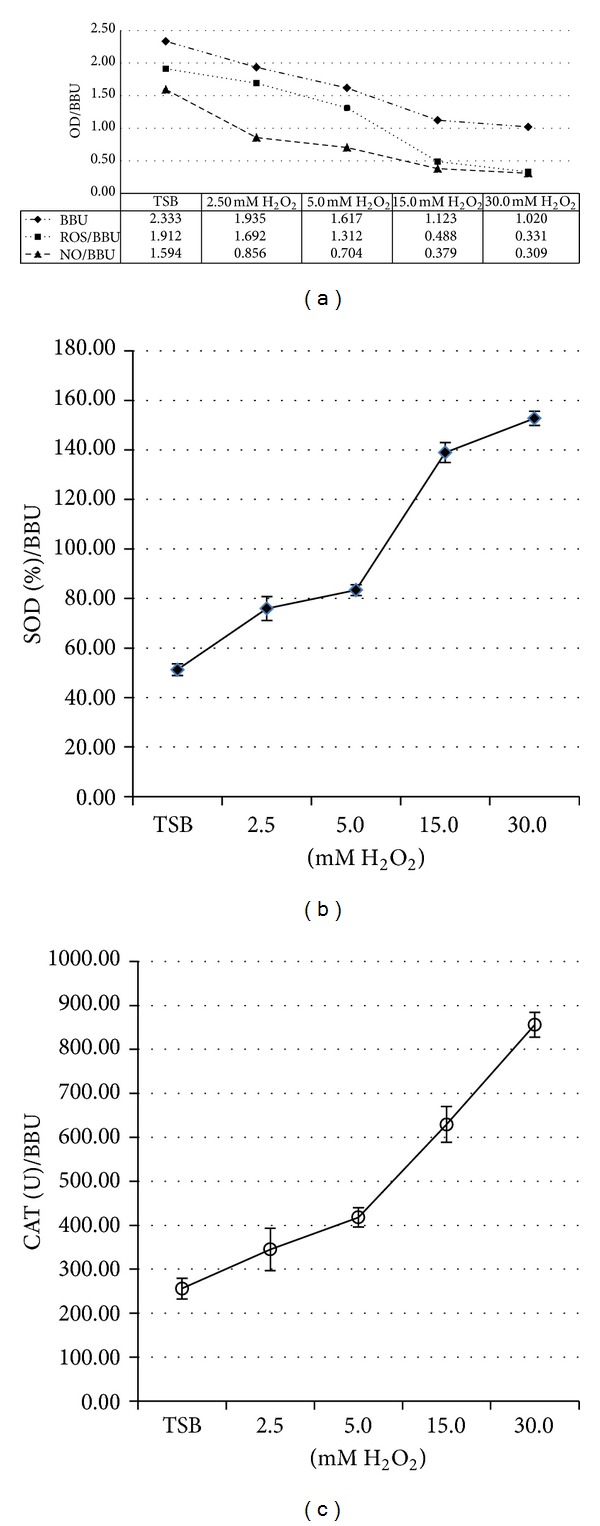
Biofilm formation with H_2_O_2_ treatment (range of 2.5 to 30 mM) of strain N° 1. (a) Biofilm biomass units (BBU) (-◆-), relation of ROS and BBU (ROS/BBU) (-■-), and NO and BBU relation (NO/BBU, nitrite expressed in *μ*M) (-▲-); (b) SOD activity (%)/BBU (-∙-); and (c) CAT (U)/BBU (-∘-). Each line shows the mean ± SEM of three independent experiments.

In order to assess the oxidative imbalance, H_2_O_2_ was added as exogenous stressor. In Figure 3, the results obtained with strain N° 1 are represented, similar results were obtained with strains N° 2 and the reference strain. H_2_O_2_ significantly reduced the biofilm BBU after 24 h of incubation and it was concentration-dependent, with less biofilm formation occurring at 30 mM. A reduction of the levels of ROS and RNI was also detected (Figure 3(a)). The H_2_O_2_ added seems to have the capacity to stimulate SOD and CAT activity in biofilms (Figures 3(b) and 3(c)). The change in the levels of antioxidant defenses was increased, as a protective response to stressful conditions due to the ROS and RNI levels.

### 3.2. Cytotoxic Effects on Vero Cells

**Figure 4:**
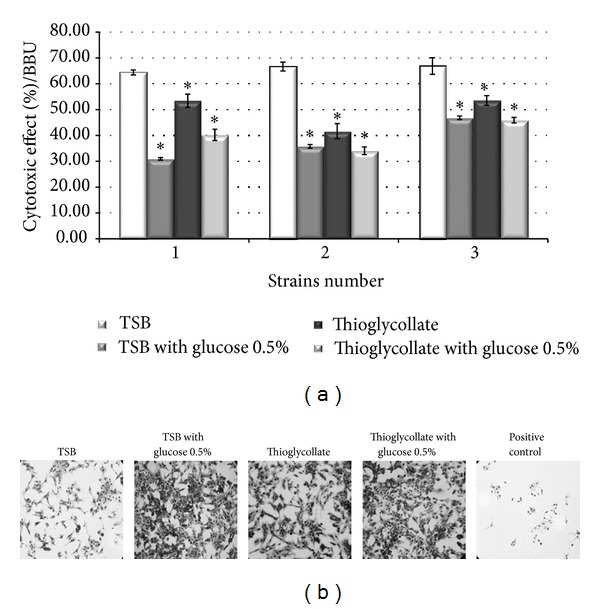
Vero cell cytotoxicity assay. (a) The percent specific cytotoxicity was determined by microscopic quantification after staining the cells. (b) Micrographs of one representative independent experiment of strain N° 1 are depicted (right). *P versus TSB < 0.01 and ^#^ *P* versus thioglycollate medium < 0.01 being considered statistically significant.

To further evaluate the potential damage induced by Stx, the percentage of cytotoxicity was evaluated on Vero cells. The results summarized in Figure 4 indicate that the Stx release was observed from the biofilms in different growing conditions, being proportional to the stress observed of biofilms and it resulted into a significant increase in cellular toxicity. However, the maximum effect of cytotoxicity was observed with the supernatant treated with H_2_O_2_.

## 4. Discussion 

 Although outbreaks of STEC infections have been primarily associated with eating undercooked ground beef, a variety of other foods have also been implicated as vehicles. Moreover cross-contamination of foods can occur in food-processing plants and during subsequent handling and preparation, resulting in a wide range of foods being implicated in outbreaks of STEC infections. The ability of bacteria to attach to and produce biofilms on surfaces may influence their persistence during manufacturing and retail, as well as their ability to cause disease, with biofilm cells often being more resistant to various stresses than their planktonic counterparts [9, 10, 24]. 

 Many investigations have disclosed that the presence of an appropriate sugar source is essential for the production of a polysaccharide matrix, with a low sugar concentration often being a limiting factor. Glucose, over a narrow concentration range, has been previously reported to increase biofilm formation [13, 25], and our results are in agreement with these results, since the clinical strain of *E. coli* O157 : H7 (N° 1) increased by 2.5-fold the biofilm formation, with other strains also leading to a higher formation, but to a lesser extent compared to strain N° 1. We also found that mannose increased the biofilms. Biofilms formed under favorable conditions may protect STEC against the sanitizers used to decontaminate and produce processing environments [10].

 Microcolony structures, due to endogenous oxidative stress, are specific sites within biofilms where enhanced genetic adaptation and evolutionary change take place [26]. In addition, Boles and Singh showed that endogenous oxidative stress in biofilms promotes antibiotic resistance and that the addition of antioxidants reduces the diversity of biofilms [27]. Recently, it was observed that the increased production of oxidative stress causes changes in the extracellular polysaccharide (EPS) in biofilms of *S. aureus* [13]. In STEC, our results demonstrated that in presence of sugars or low oxygen there was an increase in biofilms respect to basal conditions, and this could have been related to the low production of ROS and NO observed in the present investigation.

 The role of the periplasmic antioxidant enzymes of the Shiga toxin-producing *E. coli* O157 : H7 in the formation of biofilms was studied by proteomic analyses, and significantly higher expression levels of zinc superoxide dismutase and thiol peroxidase were found in STEC cells grown under biofilm conditions than these under planktonic conditions [28]. We found in STEC, that SOD and CAT levels are low under favorable conditions, because the levels of ROS are also low in these biofilm cells.

Mai-Prochnow et al. [26] have suggested that H_2_O_2_ allows to (directly or indirectly) kill a subpopulation of cells and increase in DNA damage and mutation frequency of the remaining live cells and shown that high CAT activity can prevent penetration of hydrogen peroxide into biofilms of *Pseudomonas aeruginosa* at a concentration of 50 mM. In our work, it was observed that biofilm development is influenced by the production of oxidants metabolites and the levels of antioxidant defenses, which can be variable in different environmental conditions. We found that SOD and CAT levels are low under favorable conditions, because the levels of ROS are also low in these biofilm cells. We suggest that when this balance was altered by unfavorable conditions, an increase in the ROS production induces an overproduction of cellular stress, resulting in higher levels of SOD and CAT being able to successfully detoxify the ROS generated by H_2_O_2_.

Our results show that there was release of toxin from biofilms, with this being the first report in STEC. This release was influenced by different culture conditions and a link was found between this release and the stress present in sessile cells. In conclusion, in the present study, we have observed that biofilm development was influenced by the production of oxidants (ROS and RNI) and the levels of antioxidant defenses (SOD and CAT), which may have been affected by environmental conditions and this has an effect on the release of Stx. This oxidative imbalance produced by the alteration of biofilm environment may have an important role in the pathogenesis of infections caused by *E. coli* strains that produce this toxin. In future, an improved understanding of the mechanisms involved in the release of toxins during biofilm formation would contribute to a better understanding of the pathogenesis of STEC.
